# Double Trouble on the Lower Leg—Unique Human Coinfection with *Echinococcus granulosus* and *Echinococcus multilocularis* Without Liver Involvement

**DOI:** 10.3390/pathogens14040343

**Published:** 2025-04-03

**Authors:** David Beck, Mirjana Balen Topić, Klaudija Višković, Neven Papić, Rado Žic, Mario Sviben, Tomislav Meštrović, Adrijana Baković Kovačević, Relja Beck

**Affiliations:** 1 School of Medicine, University of Zagreb, 10000 Zagreb, Croatia; dabeck025@gmail.com (D.B.); npapic@bfm.hr (N.P.); radozic123@gmail.com (R.Ž.); mario.sviben@hzjz.hr (M.S.); 2 University Hospital for Infectious Diseases “Dr. Fran Mihaljević”, 10000 Zagreb, Croatia; kviskovic@bfm.hr; 3 Faculty of Health Studies, University of Rijeka, 51000 Rijeka, Croatia; 4 Department of Plastic, Reconstructive and Aesthetic Surgery, Dubrava University Hospital, 10000 Zagreb, Croatia; 5 Microbiology Service, Parasitology Department, Croatian National Institute of Public Health, 10000 Zagreb, Croatia; 6 University Centre Varaždin, University North, 42000 Varaždin, Croatia; tmestrovic@unin.hr; 7 Institute for Health Metrics and Evaluation, University of Washington, Seattle, WA 98195, USA; 8 Department of Health Ecology, Teaching Institute of Public Health “Dr. Andrija Štampar”, 10000 Zagreb, Croatia; adrijana.bakovic@stampar.hr; 9 Department for Bacteriology and Parasitology, Croatian Veterinary Institute, 10000 Zagreb, Croatia; beck@veinst.hr

**Keywords:** *Echinococcus multilocularis*, *Echinococcus granulosus*, alveolar echinococcosis, cystic echinococcosis, coinfection, extrahepatic, muscle

## Abstract

The tapeworms *Echinococcus granulosus* and *Echinococcus multilocularis* cause two different clinical manifestations in humans: cystic echinococcosis (CE) and alveolar echinococcosis (AE), respectively. Both forms of echinococcosis manifest primarily in the liver, while other organs or tissues are less frequently affected. Simultaneous occurrence of CE and AE is extremely rare, and all previously reported patients exhibited affected livers, while simultaneous infection without liver involvement has not yet been described. Herein, we present an exclusively extrahepatic *E. granulosus* and *E. multilocularis* coinfection localized between the calf muscles of a patient. Due to progressive painful local swelling, an abscess was suspected, but there was no improvement after the administration of multiple courses of antibiotics. When imaging diagnostics suggested a parasitic origin of the two identified cystic lesions, positive serology for both species indicated a dual infection. Albendazole therapy was started, and extensive surgical excision was performed. Both species were confirmed using PCR and sequencing from intraoperative samples. The current case shows that coinfection without liver involvement can occur, even in patients from low-incidence regions, which should be considered in the differential diagnosis of patients with unusual clinical presentation.

## 1. Introduction

Human echinococcosis is a zoonotic disease caused by the metacestode stage of tapeworms from the *Echinococcus* genus. The disease can be transmitted to humans through the ingestion of embryonated parasite eggs from the soil, contaminated food or drink, or after direct contact with an infected animal [1]. In Europe, echinococcosis can be caused mostly by two species, *Echinococcus granulosus* and *Echinococcus multilocularis*, which cause two different clinical manifestations in humans: cystic echinococcosis (CE) and alveolar echinococcosis (AE), respectively. In both infections, humans serve as accidental hosts, and although the transmission routes are the same, the main hosts and thus, the geographical distribution and risk factors for infection, differ considerably, depending on the *Echinococcus* species involved [2]. The main hosts for *E. granulosus* are canines, of which the domestic dog is the most important, while the main host for *E. multilocularis* is the red fox (*Vulpes vulpes*), although other definitive hosts, such as domestic dogs, might play a substantial role in the transmission to humans [3,4]. Due to the different biology of the parasitic cysts, the clinical manifestations of infections in humans are characterized by different clinical courses and prognoses. In case of CE, a single cyst or, more rarely, several cysts gradually increase in size by concentric growth, which is usually asymptomatic. But, in rare cases, symptoms of local compression, secondary bacterial infection, or even rupture can develop. In contrast, the growth of an AE lesion is slower and more insidious, consisting of a conglomerate of many small cysts, often interspersed with necrotic tissue and calcifications, growing by lateral budding across the tissue, regardless of the anatomical boundaries—similar to a cancerous lesion [5]. In this way, human AE is usually diagnosed in late stages of the disease, and has a much worse prognosis, with sometimes even lethal outcomes.

In general, the liver serves as the primary site for the manifestation of both forms of echinococcosis. In case of CE, the liver is affected in 69–75% of cases, while the lungs represent the second most commonly involved organ, accounting for 17–22% of cases [5]. AE can more often be considered as a primary liver disease, as the liver is affected in almost all events, and extrahepatic growth is present in only 3% of cases [6]. In addition, hepatic AE lesions are more prone to continuous spread to the neighboring tissues or organs, which could be found in 34% of cases, e.g., diaphragm, retroperitoneal tissue, abdominal lymph nodes, extrahepatic vessels or ligaments and the peritoneum, and in rare cases, metastatic spread to more distant organs can be found [6].

The dual infection with *E. granulosus* and *E. multilocularis* is very rarely observed in humans. The majority of coinfected patients are reported from highly endemic areas in China [7,8]. In all cases reported, the liver was always involved, containing lesions caused by either both, or at least one, *Echinococcus* species [7].

Apart from being a rare disease, extrahepatic echinococcosis, whether primary, secondary, or as a coinfection, typically exhibits an unusual clinical presentation, which can mislead clinicians and negatively affect the diagnostic and therapeutic decisions, especially in regions with a low or extremely low incidence of disease. Delayed diagnosis, as well as inappropriate treatment, can thus have a negative impact on the course of the disease.

Dalmatia, the Mediterranean part of Croatia, has historically been an endemic area for CE. There was a decrease in the CE incidence of over 70% from the mid-1950s until late 1990s [9]. However, patients with CE are occasionally still diagnosed all around the country. On the contrary, the first evidence of infection with *E. multilocularis* was described only recently, in 2015, in red foxes [10]. Shortly thereafter, the first human case of AE was described in 2018 [11]. Since then, the incidence of human AE appears to be increasing, with most cases concentrated in the region of central continental Croatia [12]. However, both human CE and AE are rare diseases in Croatia, and an exclusively extrahepatic focus of either of these infections has not yet been reported in our patients.

Thus, we present, to the best of our knowledge, the first case of human concurrent CE and AE coinfection localized solely outside the liver, among the muscles of the calf. The surprising localization and unusual clinical course of these parasitic diseases, in a patient from a low-incidence region, make this case instructive, which may help when diagnosing similar patients in the future.

## 2. Case Description

A 71-year-old female patient came to the University Hospital for Infectious Diseases in June 2024 due to chronic symptoms present on her left lower leg, looking for a second medical opinion after several medical examinations performed by a general practice doctor, surgery specialists, and an infectious disease specialist at the local county hospital.

**Figure 1:**
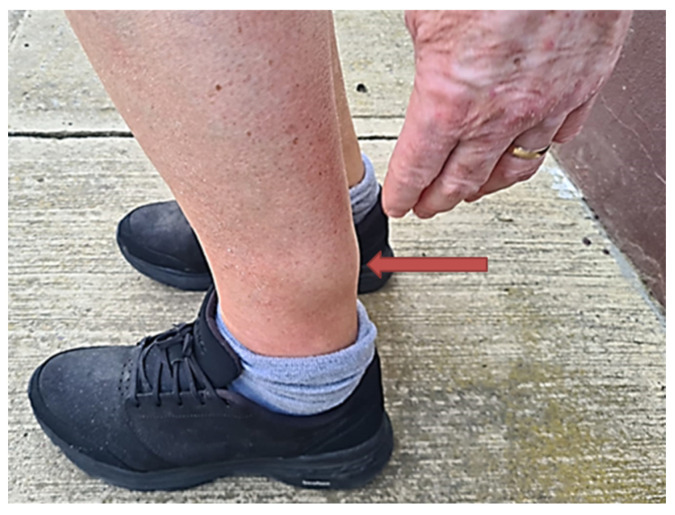
Status of the patient’s left lower leg at the early stage of the disease (slightly painful, gradually growing tumorous swelling is indicated by the red arrow).

Ten months earlier, in August 2023, a slowly progressing, increasingly painful swelling appeared on her left calf (Figure 1). Despite the absence of fever, a local infection was suspected, and the patient was treated with several courses of broad-spectrum antibiotics (ciprofloxacin, amoxicillin/clavulanic acid twice, piperacillin-tazobactam, doxycycline) during the 8-month period, including one in-hospital treatment.

Despite applied antibiotic therapies, neither significant nor permanent improvement appeared. Contrary to expectations, 6 months after the initial symptoms, in February 2024, local pain exacerbated and local status worsened, leading to surgical incision, drainage, and biopsy, which was performed at the local county hospital, under clinical suspicion of a calf abscess. According to the patient’s words, after the wound was squeezed, numerous “whitish, rubbery balls, up to 1–1.5 cm in diameter”, were expelled through the incision wound. Although they were not sent for microscopic analysis, the histopathology of the biopsy sample revealed fragments of amorphous, eosinophilic material, without parasitic elements. Afterwards, a post incisional fistula developed, through which “whitish membranes” would occasionally pour out. At that time, the patient’s laboratory findings were unremarkable, except of a slight, transitory eosinophilia of 700 eosinophils per µL of peripheral blood, recorded shortly after surgical intervention, which spontaneously withdrew. The patient had a history of arterial hypertension, hyperlipidemia, chronic gastritis, and hypothyroidism, and in 1990, underwent neurosurgical removal of a meningioma. Due to those chronic diseases, she regularly received appropriate therapy. Additionally, for a couple of years, a simplex liver cyst of 2 cm in diameter, multiple small simplex cysts of the kidneys, and a Backer’s cyst in the left popliteal region were known, showing no progression on the control imaging.

The patient was a retired woman who lived in a house with a garden, in a small town in eastern continental Croatia (45°35′ N 18°28′ E / 45.59° N 18.46° E), who had owned a dog for over a year, denied having contact with other animals or a forest environment, and had not travelled abroad within the last 15 years.

The examination at the University Hospital revealed slightly elevated blood pressure of 140/90 mm/Hg, and a 2.5 cm long dry incision wound, without local signs of inflammation and without any secretion after pressure, situated on the lateral, middle to distal third of the left calf. Other physical findings were normal.

**Figure 2:**
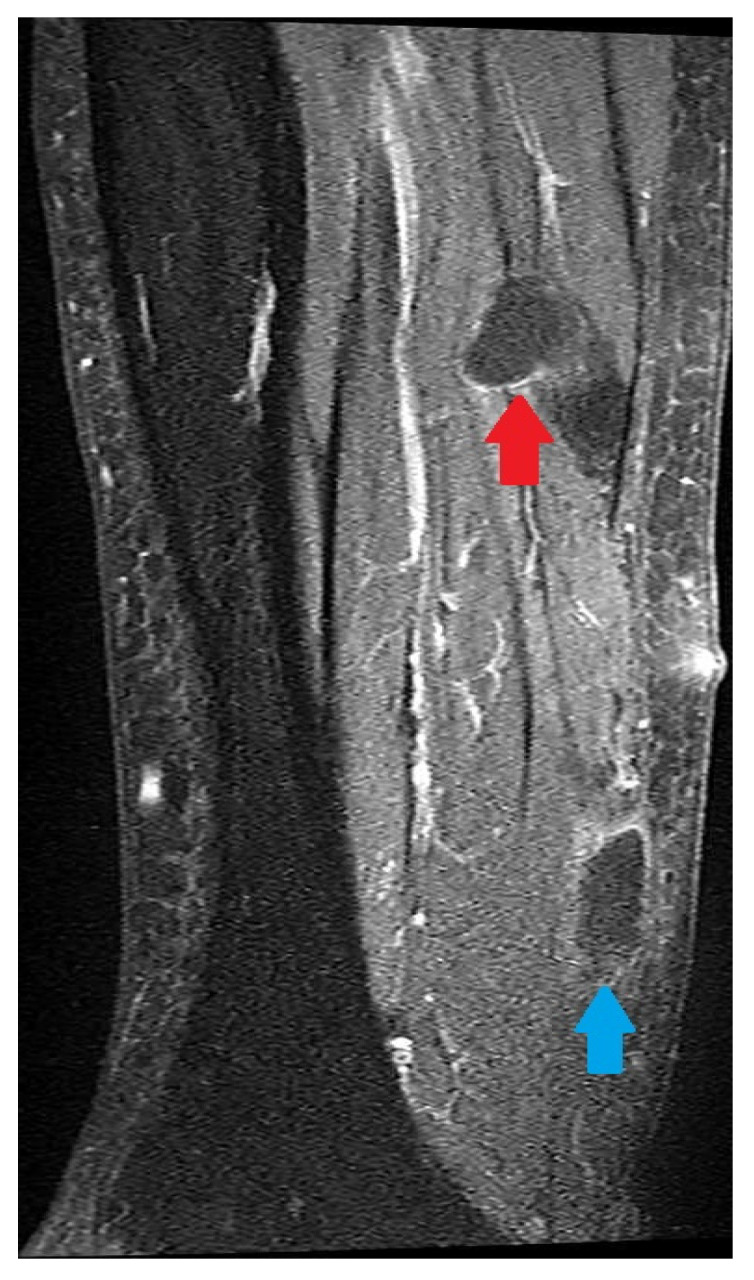
A postcontrast magnetic resonance imaging (MRI) scan of the patient’s left calf, performed on 18 June 2024, after surgical incision, revealed two focal lesions with different morphological features (red and blue arrows). The first one was situated in the proximal part of the calf, inside the soleus muscle. At the postcontrast T1 WI MRI scan, the lesion was dominantly hypointensive compared with the surrounding muscle with discreet postcontrast ring enhancement, with defined contours (red arrow). The second lesion was an extramuscular, subfascial collection of dense fluid, located in the distal third of the left calf, near the dorsal part of the soleus muscle and the dorsal part of the Achilles tendon. On a postcontrast T1 WI MRI scan, the lesion was hypointense with postcontrast ring enhancement and no clear border (blue arrow).

Magnetic resonance imaging (MRI) of the left lower leg was indicated on that occasion, and it was performed in June 2024. It showed two cystic/solid morphologically diverse lesions, mostly hypointense with discreet postcontrast ring enhancement (Figure 2). Multi-slice computer tomography (MSCT) of the lower legs, captured one month earlier, was in accordance with the MRI finding. Although MRI displays better contrast resolution than do the MSCT scans, in our patient, the two described lesions were not distinctive enough, so a CE and AE coinfection could not have been suspected at this point. MRI of the head in February 2024, chest X-rays in May 2024, and MSCT of the abdomen and pelvis in June 2024 showed no suspicious parasitic lesions, which was in accordance with the absence of any additional focal symptoms in our patient. In May 2024, serological testing performed 9 months after the onset of clinical symptoms revealed positive enzyme linked immunoassay (EIA) results for the semiquantitative determination of the IgG class of antibodies against *Echinococcus* spp. (test NovaLisa *Echinococcus* IgG ELISA, manufacturer Gold standard diagnostics Frankfurt GmbH), and positive *E. multilocularis* semiquantitative detection of IgG antibodies against Em2 and Em18 specific antigens (*E. multilocularis* ELISA test, manufacturer Bordier Affinity Products, Crissier, Switzerland), accompanied by a positive confirmatory qualitative test *(Echinococcus* western blot IgG, manufacturer LDBIO Diagnostics, Lyon, France) for serological diagnostics of alveolar and hydatid echinococcosis. Since bands common to both species were found, the dual infection was suspected for the first time in June 2024.

Due to positive serology, molecular diagnostics were performed retrospectively on the previously stored surgical paraffin block collected in February 2024. The presence of *E. granulosus* was confirmed by positive PCR and sequencing of the *COI* gene [13], while specific PCRs for *E. multilocularis* that amplify *nad1* [14] and the *12S rRNA* genes [15] were negative in that sample.

Since the patient’s first visit to the University Hospital for Infectious Diseases in June 2024, a continuous antiparasitic therapy with albendazole (2 × 400 mg tablets per day) was started, and radical surgical excision was advised.

Due to comprehensive and complicated relationship between the parasitic lesions and local anatomic structures, surgery was performed by a plastic surgeon in September 2024. On that occasion, half of the m. soleus, subcutaneous tissue with the fascia of the m. gastrocnemius, together with the skin and subcutaneous tissue of the distal calf, were removed. Nerves and blood vessels were preserved. The surgical site was rinsed with disinfecting sodium hypochlorite and hypochlorous acid solution, and profusely soaked with hypertonic saline for 30 min, before closing.

Histopathological examination of multiple intraoperative samples from both locations (1. tissue of m. soleus with cyst formation, and 2. tumorous/cystic tissue with skin and muscle from the distal calf) revealed nonspecific granulations. They contained acellular eosinophilic masses which corresponded to the hydatid cyst walls. Elements of parasites were not found by this method, which made the involved *Echinococcus* species impossible to determine.

However, two macroscopically different types of samples were sent for molecular diagnostic testing.

**Figure 3:**
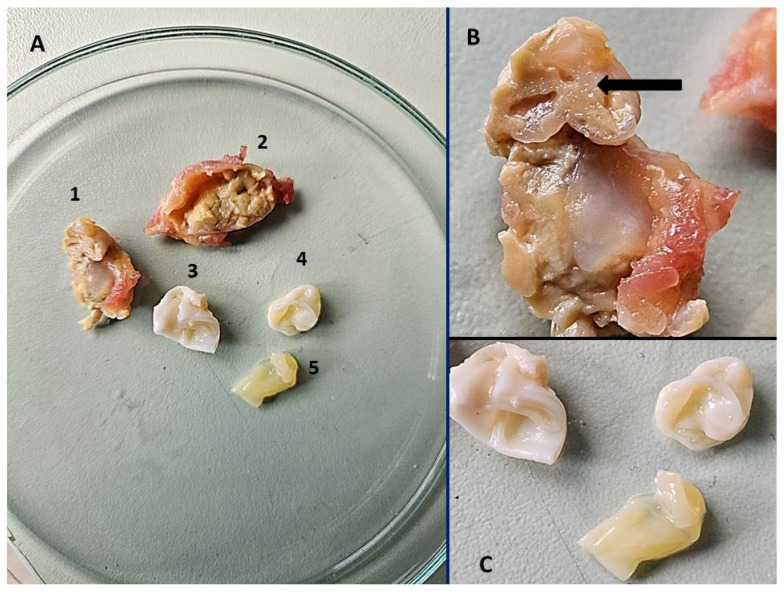
Cysts and tissue sections (1–5) removed from the patient’s left calf during surgery in September 2024 which were used in molecular diagnostic testing (**A**). A large portion of the sample that tested positive for *E. multilocularis* labeled with a black arrow (sample 1) (**B**). Cysts positive for *E. granulosus* (samples 3–5) (**C**).

Both *Echinococcus* species were confirmed from morphologically different cysts collected during the surgery (Figure 3). Sequencing of the *COI* [13], *nad1* [14], and *12S rRNA* genes [15] and comparison with existing sequences in GenBank using BLAST (version 2.16.0.) revealed *E. multilocularis* in sample number 1 and *E. granulosus sensu stricto* from the cysts labeled 3, 4, and 5 using *COI* [13]. Furthermore, the sequenced *ATP6* gene [16,17] from the cysts labeled 3, 4, and 5 supported its classification as *E. granulosus* genotype G1. The sequences are deposited in GenBank and are available under the following accession numbers: PV188742 (*E. multilocularis*, *COI*), PV197949 (*E. multilocularis*, *12S rRNA*), PV189156 (*E. granulosus*, *COI*), and PV224466 *E. granulosus*, *ATP6*).

The postoperative course was uneventful, and the patient recovered well. Only nonsignificant elevation of transaminases due to continuous albendazole therapy appeared postoperatively, which did not require an interruption of therapy. For greater clarity, the timeline of diagnosis and treatment is shown in Table 1.

## 3. Discussion

The described patient presents, to the best of our knowledge, the first case of human *E. granulosus* and *E. multilocularis* coinfection localized exclusively in the muscle and soft tissue, without liver involvement. Although capable of infecting virtually any organ or tissue in the human body, due to anatomic reasons, the attack rate for both CE and AE is the highest in the liver, which serves as the filter for venous portal blood flowing from the gut. The mechanism of primary extrahepatic infection is currently still being hypothesized. An important factor could be the ability of oncospheres to directly invade the lymphatic vessels and bypass the liver [18]. The pathogenesis of coinfection also remains elusive, since it is believed that there exist some mechanisms which could stop the simultaneous growth of both species [19]. However, musculoskeletal involvement by each type of echinococcosis in humans is rare. In case of CE, it is estimated to account for 0.5–4% of cases [20]. So far, the lesions of CE have been described in hamstrings and adductor muscles [21,22,23]; the vastus lateralis [24]; the infraspinatus muscle of the shoulder [25], suprapubic, pubic bone, and left pectineus muscle [26]; the calf muscles [20]; the gluteal region [27]; and the psoas muscle [28]. All of these cases have shown to be primary localizations of CE, with an exception of recently reported pelvis muscle involvement, developed after retrovesical hydatid cyst surgery, which was possibly iatrogenic [26]. Primary cases of extrahepatic soft tissue AE seem to be even less frequent than those of CE [18,29,30]. The extrahepatic finding of AE alone, without liver involvement, is extremely rare. It has been described in the spleen [18], the psoas muscle [29,31], the parotid gland [32], and in the lumbar spine with spondylodiscitis—later with a lethal outcome [33]. Cases of CE and AE coinfection in humans can be extremely rarely found in the literature. They are all reported from highly endemic areas such as the Tibetan plateau in China, where high environmental factors favor coinfections [7,8]. As far as we are aware, in every described case of coinfection up until now, the liver was involved, either affected by both *Echinococcus* species, or in conjunction with another organ affected by a second species [34,35,36,37].

Although the exact incidence of human CE in Croatia is not known, it can be estimated as low, according to clinical experience at our University Hospital Center, to which all severe or unusual clinical cases gravitate. However, the presence of human AE in Croatia has been only recently recognized, but the number of diagnosed patients seems to increase continuously, reaching 15 by December 2024 [38]. The region with the highest incidence of 2.94 cases per 100,000 inhabitants in 2022 has been identified in central continental Croatia [12]. So far, the liver has been identified as the affected organ in all Croatian AE patients. Interestingly, the patient in this study lives in the east Croatia region, has no direct contact with foxes, and has no obvious epidemiologic risk for AE.

It is difficult to guess how our patient became infected with both species of *Echinococcus*, as the reservoirs are usually different, although dogs and golden jackals can harbor both species of *Echinococcus* as main hosts. Since our patient, like many of her neighbors, had a dog, we hypothesize that the dog could be the source of her infection. Infection of dogs with *E. multilocularis* has been demonstrated experimentally [39], and “being a dog owner” has been recognized as a risk factor for *E. multilocularis* infection in humans [1,40]. The same is true for the golden jackal [41], an opportunistic, invasive canid that approaches and invades suburban areas to live near humans [42]. Since the golden jackal has become the dominant wild canid in the region where the patient lives, and an infection with *E. multilocularis* has been found in this species in Croatia [43], one could assume that the source of the double infection in our patient could also be the golden jackal. As contact with the definitive host is unlikely, we assume that the patient became infected via contaminated fruit or vegetables.

In addition to the scarce exposure, which may be completely absent in some cases of human echinococcosis [32], our patient did not have significant comorbidities such as diabetes mellitus or liver cirrhosis that seem to favor extrahepatic involvement [18,33].

The bizarre clinical presentation also misled the clinicians involved, contributing to a 10-month delay in diagnosis and a 13-month delay of surgical treatment. Due to the different biology of the metacestode stage between *E. granulosus* and *E. multilocularis* (concentric growth vs. lateral budding, faster vs. slow insidious growth, respectively) [44], the symptoms of progressive, painful swelling of the calf in our patient were most likely caused by the *E. granulosus* lesion. Since the “whitish, rubbery balls” expelled at the first incision were not recognized as parasitic cysts, and since the initial histopathological, MSCT and MRI findings yielded non-specific results, after *E. granulosus* infection had been confirmed by the retrospective molecular diagnosis of the initial biopsy sample, the first indication of a double infection was provided by serology test results. Serology has proven to be a useful tool in the diagnosis of human echinococcosis, but its results cannot always be used as a definitive diagnosis. The sensitivity of a serological test depends on the stage of the cyst, its size, the immunity of the host, local epidemiology, and the test method used. Additionally, it should be kept in mind that the manufacturer’s declared sensitivity of a serological test could significantly decrease in the case of extrahepatic locations of parasitic lesions, and also the results could be confusing, suggesting *Echinococcus* coinfections due to the possibility of cross reactivity [45]. However, in our patient, positive serology results for both *Echinococcus* species suggested radical excision of both lesions, from which, in the sample taken from the distal cyst, *E. multilocularis* was clearly proven by sequencing the *nad1*, *COI*, and *12SrRNA* genes, while *E. granulosus sensu stricto* was confirmed in the three samples collected from the proximal cyst by sequencing the *COI* and *ATP6* genes.

As for hepatic localization, MSCT, and especially MRI, have shown to be even more important tools for diagnosing extrahepatic CE and AE, including the location in the musculoskeletal system [46,47]. But, despite the high resolution of their scans, the results of diagnostic imaging may show uncharacteristic, unusual, and in some cases, even bizarre findings. Although common radiological features for liver infections for both CE and AE have already been described [8,30,37], even in case of intrahepatic CE and AE coinfections, the radiological findings can become skewed [35]. When it comes to extrahepatic infections of both AE and CE, radiological signs become less specific and depend more on the localization of the infection [21,25,29,30,32]. Due to the rarity of extrahepatic infections, the variety of anatomical sites that may be affected, and the uniqueness of the morphology of the lesions, no characteristic sign has been identified as a likely feature of musculoskeletal echinococcosis, and it is impossible to establish a clinically useful classification system for extrahepatic disease. This is especially true for AE, which is often mistaken for a malignant tumor due to its invasive growth, regardless of the anatomic barriers, even when found in the liver. The MSCT and MRI scans of the two lesions from May and June 2024, showed a cystic appearance with post-contrast enhancement. One lesion displayed sharp margins, while the other exhibited irregular margins. Both *Echinococcus* species rarely affect muscles and may have different imaging features, depending on the stage of disease, which can lead to diagnostic difficulties. However, echinococcosis should always be considered in the differential diagnosis of cystic lesions.

With the aim of definitively ruling out the possibility of a primary intra-abdominal localization of the infection, we retrospectively analyzed the patient’s abdominal ultrasound examinations from 2019 and 2022 and compared them with those from 2024. The size and ultrasound morphology of all described cysts in the liver and kidneys remained unchanged. Since the cysts in the liver and kidneys did not change over the five-year period and did not show the “double line” sign or fine echoes within the cysts (which may represent “hydatid sand”), we concluded that they could not be classified as hydatid cysts. Furthermore, their morphology did not correspond to the typical appearance of AE, which often occurs as a primary tumor-like lesion. In our patient, both lesions looked similar on MSCT and MRI scans, and based on their morphology, coinfection could not have been suspected, which emphasizes the necessity of applying other direct and indirect diagnostic methods in unusual cases.

## 4. Conclusions

Our case shows that even in patients with no apparent risk factors, living outside highly endemic regions, *E. granulosus* and *E. multilocularis* coinfection can develop, manifesting solely at an extrahepatic location, which should be taken into differential-diagnostic consideration in patients with unusual clinical presentations. In our case, serology has proven to be a useful diagnostic tool for CE and AE, even without liver involvement. Furthermore, this case emphasizes that in the event of multiple lesions at any location, it is necessary to perform multiple samplings of each lesion and conduct molecular analysis of several samples, since histopathology could reveal nonspecific results. A comprehensive multidisciplinary approach to the patient is essential in resolving such a case. In regions with low incidence of human echinococcosis, additional effort should be focused on the education of medical professionals with the aim of raising the level of clinical suspicion in order to accelerate the diagnostic process and improve disease outcomes.

## Figures and Tables

**Table 1:** Timeline of diagnostic testing and treatment from initial suspicion of an abscess to objective confirmation of coinfection with *E. granulosus* and *E. multilocularis*.

	August 2023	February 2024	March 2024	May 2024	June 2024	September 2024
Surgery		Incision and biopsy on the lesion of the calf				Surgical excision of lessions of the calf
Radiology		MRI of the head		MSCT of the calf Chest X-ray	MRI of the leg; MSCT of abdomen and pelvis	
Histopathology			Histopathologically described as eosinophilic amorphous material			
Molecular analysis					PCR and sequencing from paraffin blocks collected in February 2024 confirmed *E. granulosus*	PCR and sequencing, confirmed *E. granulosus* and *E. multilocularis*
Serology				EIA, WB positive for *E. granulosus* and *E. multilocularis*		
Treatment	Multiple antibiotic courses due to suspected abscess				Albendazol treatment 2 × 400 mg	
					

The red/blue colors signify the time frame for antibiotic start and stop as well as for antiparasitic treatment, respectively.

## Data Availability

The datasets from the current study are available upon request to the corresponding author.
